# The Inner Complexities of Multimodal Medical Data: Bitmap-Based 3D Printing for Surgical Planning Using Dynamic Physiology

**DOI:** 10.1089/3dp.2022.0265

**Published:** 2023-10-10

**Authors:** Nicholas M. Jacobson, Jane Brusilovsky, Robert Ducey, Nicholas V. Stence, Alex J. Barker, Max B. Mitchell, Lawrence Smith, Robert MacCurdy, James C. Weaver

**Affiliations:** ^1^ School of Engineering, Design, and Computation—Inworks Innovation Initiative, University of Colorado Anschutz Medical Campus, Aurora, Colorado, USA.; ^2^ LAIKA Animation Studio, Portland, Oregon, USA.; ^3^ School of Medicine, University of Colorado Anschutz Medical Campus, Aurora, Colorado, USA.; ^4^ Children's Hospital Colorado, Heart Institute and Advanced Imaging Lab, Aurora, Colorado.; ^5^ School of Engineering, University of Colorado Boulder, Boulder, Colorado, USA.; ^6^ Wyss Institute for Biologically Inspired Engineering, Harvard University, Cambridge, Massachusetts, USA.

**Keywords:** bitmap printing, 3D printing, biomedical imaging, physiology

## Abstract

Motivated by the need to develop more informative and data-rich patient-specific presurgical planning models, we present a high-resolution method that enables the tangible replication of multimodal medical data. By leveraging voxel-level control of multimaterial three-dimensional (3D) printing, our method allows for the digital integration of disparate medical data types, such as functional magnetic resonance imaging, tractography, and four-dimensional flow, overlaid upon traditional magnetic resonance imaging and computed tomography data. While permitting the explicit translation of multimodal medical data into physical objects, this approach also bypasses the need to process data into mesh-based boundary representations, alleviating the potential loss and remodeling of information. After evaluating the optical characteristics of test specimens generated with our correlative data-driven method, we culminate with multimodal real-world 3D-printed examples, thus highlighting current and potential applications for improved surgical planning, communication, and clinical decision-making through this approach.


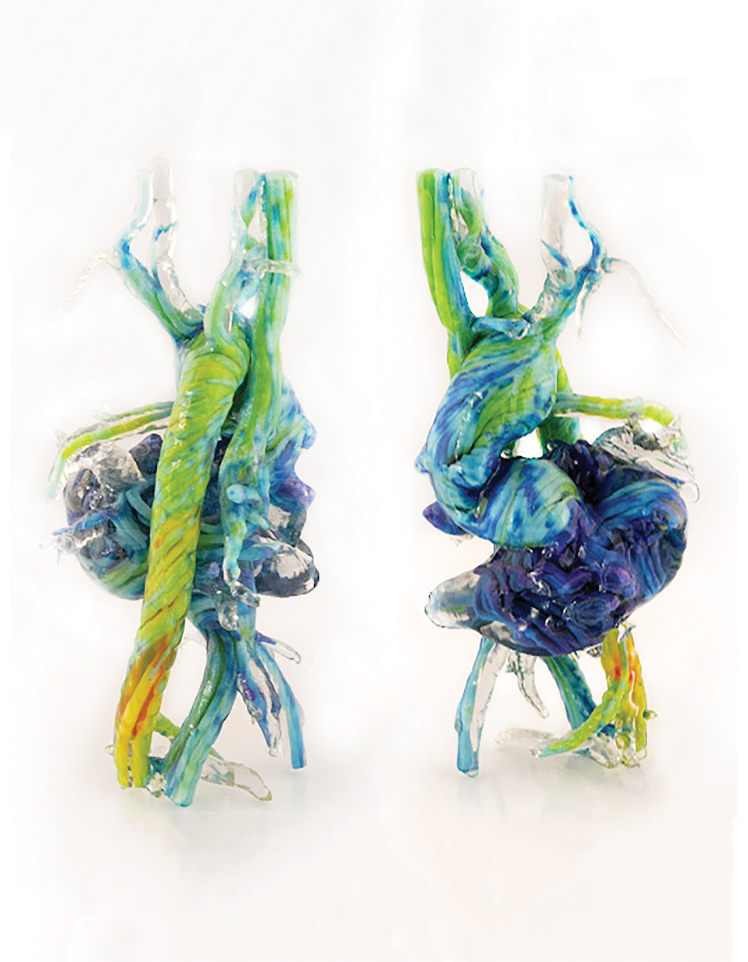


## Introduction

Medicine has long relied on tangible objects to provide clinical insights about disease states, which can be traced back thousands of years to the use of stone and clay models.^1^ However, since the discovery of X-rays by Wilhelm Röntgen in 1895, diagnostic anatomical representations have been dominated by the use of penetrating radiation-based two-dimensional (2D) medical imaging technologies. Modern approaches to diagnostic medicine now rely on a wide range of digital data obtained from magnetic resonance imaging (MRI), computed tomography (CT), positron emission tomography, and ultrasound. Through the generation of 3D representations, these approaches map, process, and represent data with the aim of allowing doctors to gather insights and form diagnoses through sequential multimodal correlative observations.

As these technologies have continued to evolve, medical imaging has since separated into multiple subspecialties, each focused on a discrete biological function within a distinct imaging modality. These approaches, which combine the use of application-specific imaging approaches and data-processing technologies, can reveal levels of anatomical detail not previously attainable. For example, cardiovascular magnetic resonance (CMR) has become the only method for the detection of heart muscle scarring,^2^ whereas coronary computed tomography angiography best shows the perfusion of cardiac vessels.^3^ The gap between medical imaging specialties has been further institutionalized and exacerbated by divergent tools, methods, and conceptual frameworks surrounding these distinct imaging modalities.

As a result, surgical planning frequently requires the sequential and chronological review of multiple sources of discrete 2D imaging data in preparation for invasive surgical interventions. The collected imaging data are typically viewed on 2D computer screens, thus requiring significant visuospatial memory from the user. The presurgical data review process has thus become increasingly inefficient, and as such, does not provide a holistic visualization of the data, a factor that has been deemed critical for informed surgical preparation.^4^ This worrisome trajectory ignores the biggest promise of the information revolution: increased clarity, communication, connectivity, and interoperability.

In recent years, three-dimensional (3D)-printed models have emerged as valuable tangible diagnostic tools for surgical planning and have been shown to reduce operating time and surgical complications.^5^ Although conventional screen-based media visualizations are known to be effective, it has been argued that physical manifestations of data can leverage active and spatial perception skills, enabling a more comprehensive understanding of presented information in an inherently intuitive manner.^6–8^ In addition to relieving the burden on visuospatial memory, 3D models have been shown to more accurately portray scale and proportion, a critical element to biomedical applications.^9,10^ Despite these advantages, the most commonly employed additive manufacturing workflows, which rely on the use of mesh-based data, are fundamentally limited by an assembly paradigm that dates back to the Industrial Revolution, reinforced by digital modeling software, which renders printed objects as solid, homogeneous, and isotropic approximations of their source data.

As a result of these limitations, 3D printing for presurgical planning has been traditionally limited to CT-derived boney structures (excluding the comparatively lower density trabecular bone networks) and gross morphological descriptions of complex organs from MRI data, which have been shown to often lack critical anatomical details.^11^ When considering soft tissue applications, the interior of the region of interest typically consists of multiple tissue structures that combine with biological dynamics to provide diagnostic physiological markers. As such, volumetric information and dynamic physiological data are critical to understanding the potential for interventional complications, and current modeling and 3D printing methods, unfortunately, do not reflect this complexity.

In recent years, an increased clinical interest has emerged in transcending morphological structures (form) to include physiological data (function) in preparation for surgical cases, as evidenced by the increase in software focused on visualizing dynamic medical data.^12,13^ A subfield of Radiology called medical imaging computing (MIC) has grown, creating new techniques and forms of representation that derive the dynamic components of living matter from standard MRI and CT through advanced computational algorithms.^14^ MIC focuses on extrapolating physiological data from diagnostic images through advanced data processing techniques, including four-dimensional (4D) flow cardiovascular magnetic resonance (4DCMR), functional magnetic resonance imaging (fMRI), diffusion weighted imaging (DWI), diffusion tensor imaging (DTI), and stereoelectroencephalography.^14^

Attempts at 3D printing these complex data sets have produced gross approximations with the limited translation of the data into a printed form, despite showing clinical benefit.^15,16^ These limitations exist because the predominant 3D printing methods utilize a surface mesh-based paradigm, which makes the compositing of data challenging due to the need for intensive Boolean operations. Additionally, many commercially available additive manufacturing methods are incapable of capturing the level of special fidelity, soft tissue differentiation, and spatial/contrast resolution required to comprehensively translate computational data sets into 3D-printed tangible models. Since the emergence of 3D printing as a new diagnostic tool for presurgical planning, it has not yet fully realized the extent of printable data from computational imaging technologies.^17,18^ The ability to successfully do so, however, could expand the use of diagnostic physical representations of patient-specific anatomy to include integrated physiological data, thus creating new opportunities for improving patient care in a much broader range of complex and challenging surgical cases.

In contrast to the surface mesh-based methods described above, in this study, we present a 3D printing workflow that utilizes voxel-based bitmap printing technologies to collate disparate biomedical imaging methods and data into one unified diagnostic tool. Our approach renegotiates boundaries, fills gaps, and facilitates overlaps between disconnected medical imaging technologies to provide a physician with a curated, concise, and coherent physical model that integrates all available data. By creating this new tangible user interface, we are applying a wide range of new technologies to reclaim and bring over 1000 years of medical history and training into the 21st century. In doing so, these correlative data visualization approaches are anticipated to further reduce operating times, improve postsurgical outcomes, and most importantly, allow for more complex and challenging operations to be confidently performed.

In the present study, we have explored two examples that leverage a wide range of different advanced MIC technologies, including CT, MRI, cardiac 4D-MRI, and neurological tractography with fMRI, and blood oxygen level-dependent (BOLD) MRI. Using these diverse independent imaging data types, we present a series of methods for extracting, fusing, and editing MIC data into bitmap-based 3D-printed models and evaluate their potential for specific clinical applications:

1. Neuroimaging: Neuroimaging is an integral prerequisite for neurosurgical procedures since most involve intricate, minute anatomical structures that cannot be outwardly observed.^19^ When planning to resect a brain tumor, for example, neurosurgeons must consider both brain anatomy and function in different areas of the gray matter. While previous attempts have been made to represent fMRI data through 3D printing, these models have primarily been limited to opaque surface-colored solid models or have been used to visualize individual isolated elements, which disregard their spatial relationships to other anatomical features.^20,21^ In a recent effort to address some of these data resolution-related limitations, the successful 3D printing of high-resolution diffusion tractography data using voxel printing methods as defined by Bader et al.^22^ has been successfully demonstrated. To further improve the utility of these high-resolution and single-data source anatomical models, the methods described in our present study integrate fMRI, tractography, and T1 MRI data and are curated for specific clinical applications.^20,21^
2. CMR: 4DCMR has enabled a more comprehensive assessment of pulsatile blood flow through cavities of the heart and the great vessels. 4DCMR refers to phase-contrast CMR with flow-encoding in all three spatial directions, as a function of time, along the cardiac cycle (3D+time = 4D). Developed through collaborations with physicists, physicians, and biomedical engineers, 4DCMR enables a wide variety of options for visualization and quantification of flow, ranging from primary metrics, such as flow volume and peak velocity, to more advanced features such as the estimation of hemodynamic effects at the vessel wall and myocardium, or the visualization of flow pathways in the heart and great vessels. While previous attempts to visualize these processes using 3D printing-based approaches have employed computational fluid dynamics to simulate blood flow,^23^ these attempts have utilized Standard Tessellation Language-based modeling workflows, which as mentioned in the previous section, provide only a limited approximation of the inherently complex source data.

As demonstrated through these two case studies, our methods for creating tangible models of multimodal medical imaging data through bitmap-based 3D printing enable the direct, yet curated, digital manufacturing of numerous dynamic and computationally derived medical data sets. These methods and their various applications can thus unify numerous discrete medical imaging modalities of morphological and physiological data into one coherent, comprehensive, and concise physical object for use in presurgical planning.

## Materials and Methods

**FIG. 1.**
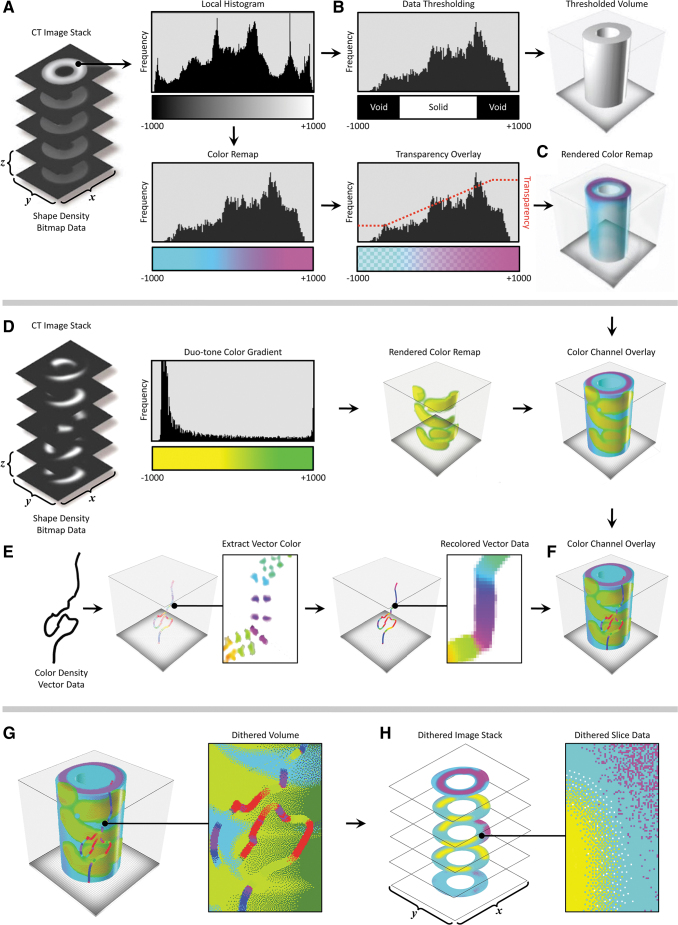
General workflow for the conversion and compositing of multimodal data sets into 3D-printed tangible objects. For a given data set **(A)**, a volume rendering is computed directly from the image-derived intensity values **(B)**. User-defined colors are then placed at specific intensity values to remap the volume rendering, and a secondary channel is modified to define the transparency of the remapped intensity values **(C)**. Additional image-based data are processed similarly to and overlaid upon **(C)** where the additional color data can be specified (if desired) to supersede the previous model voxel color transparency, and intensity values **(D)**. Color density vector data are deconstructed to extract color data stored in vertices. The imported mesh is then filled with voxels, where each voxel inherits the color of the nearest vertex **(E)**. **(F)** The resulting vector-based data are overlaid with the previous composited data generated in **(C)** and supersede the color data similar to **(D)**. To generate per-pixel material information, the volume-rendered model is dithered to reduce the colors in the model to those available within the 3D printer **(G)**. **(H)** Finally, the dithered volume rendering is sliced to the requirements of the 3D printer. 3D, three-dimensional.

An overview of our methodology is shown in Figure 1. Digital Imaging and Communications in Medicine (DICOM) data were acquired from MRI and CT instruments, providing the morphological and base data from which functional information was computationally derived. All data sets were acquired from living patients, during one or more imaging sessions, and all the resulting digital models were subsequently co-registered. Color-encoded mesh data derived from the different imaging sessions, and processed across multiple software programs, utilized a common coordinate system, guaranteeing data alignment across platforms. DICOM files were first processed with the open-source 3D Slicer software package^24,25^ and window/level adjustments, and histogram processing was completed similar to that described by Hosny et al.^24,25^

In our data processing workflow, a voxel field was created, which defined the print volume bounding box voxel resolution and channel descriptions based on those derived from the source data. A multichannel voxel model data structure was then used to convert scalar values into volumetric representations with graded material properties.

In this multistep process, a thresholding technique was first employed to construct a volumetric model by setting the geometric channel to solid if a voxel within the field was occupied by the computed imported data, and voxels within the field not containing data were set to void (Fig. 1A, B). A second channel, the color channel, assigned the color material mixing ratio for each solid voxel. Color mixing ratios were derived from the imported data, similar to Bader et al.,^22^ which included image-based data sets, such as MRI and CT, and point clouds derived from dynamic physiological information (Fig. 1D–F). When compositing multiple imported data types, one or both channels were overlaid in a hierarchical structure. The resulting composite voxel model was then dithered into colored droplet deposition descriptions to match colored resins used by the multimaterial inkjet-based (polyjet) 3D printer (Stratasys J750) and saved to a 32-bit raster graphic format (Fig. 1G, H).

**FIG. 2.**
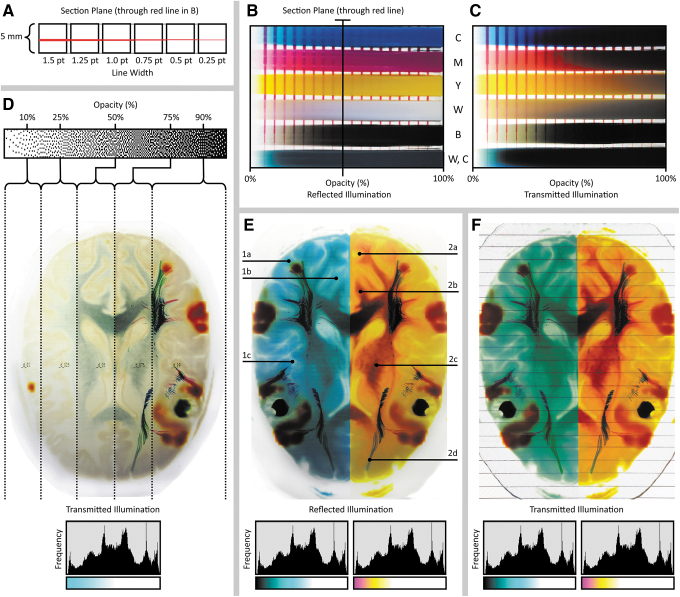
Variability in optical transparency as a function of pigment composition and data compositing resin mixing ratios. **(A)** Cross-sectional diagram of the **(B, C)** material coupons demonstrating the variability in opacity (decreasing clear resin fraction, from *left* to *right*) of different colored resins due to their unique pigment compositions (C: *Cyan*, M: *Magenta*, Y: *Yellow*, B: *Black,* W: *White*, C: *Clear*). From these data, it is apparent that translucency characteristics are not uniform across the different colored resins, can vary significantly depending on the direction of ambient lighting (reflected vs. transmitted illumination), and are not linearly related to material mixing ratios. **(D)** A representative single data slice demonstrates the different optical effects that can be generated via different material mixing ratios produced through material dithering. **(E, F)** Comparison of identical digital models 3D printed using either *Black*/*Cyan*/*Clear* (*left*) or *Magenta*/*Yellow*/*Clear* (*right*) material droplets of opaque and transparent material and photographed via either reflected **(E)** or transmitted **(F)** illumination. The backlit image shown in **(F)** contains an underlying array of parallel *black lines*, which was used to demonstrate the variation in transparency across the material mixing combinations of this single-layer bitmap print. The resulting dramatic differences in optical effects can be leveraged to preferentially reveal specific anatomical details of interest; for example, (1a) inferior temporal gyrus; (1b) cuneus; (1c) posterior limb of the internal capsule; (2a) occipital horn of the lateral ventricle; (2b) cuneus; (2c) globus pallidus; (2d) medial frontal gyrus **(E)**.

During the data processing step, several aspects of the printer hardware must be taken into consideration to obtain the desired result. For example, commercially available colored polyjet materials exhibit varying levels of translucency (Fig. 2). As noted in Bader et al.,^22^ colored objects with a material mixing ratio of more than 70% clear showed variability in translucency (Fig. 2B), whereas colored objects in the 70–95% range provide a greater level of control over subtle differences in transparency (Fig. 2C). This phenomenon must be taken into account for the visualization of volumetric data since a linear mapping from material information to material mixing will not yield linear changes in perceivable transparency or translucency.

By leveraging this tunability in compositionally specific optical transparency, our method is capable of showing embedded features within objects that exhibit variation in color to indicate differences in tissue densities revealed through CT or MR-based imaging techniques. To effectively visualize the features of interest in a case-specific manner, several key aspects of the data must be taken into consideration when assigning material-mixing ratios during data compositing and model printing. For example, to ensure visual clarity of opaque embedded objects, the segmented background volume must remain transparent, while still maintaining the color saturation required to convey spatial relationships within the differentiated soft tissues. This effect can be achieved by adjusting the ratios of clear to colored materials to produce predictable, visually coherent, results (Fig. 2D–F). However, due to the nature of the dithering process, refining the predictability of matching the computer rendering to a 3D bitmap-printed object can be nonintuitive (Fig. 2B, C), and as such, is an area of ongoing research.

Once the different features of interest have been composited within the 3D-printable volume, the volume was sliced into a an image stack using a custom code to generate full-color PNG files in concordance with the requirements for “Voxel Printing” on a Stratasys j750 3D printer.^26^ VeroClear (RGD810) was used as the transparent material, whereas VeroPureWhite (RGD837), VeroBlackPlus (RGD875), VeroYellow (RGD836), VeroCyan (RGD841), and VeroMagenta (RGD851) were used to depict the colored regions.

## Results

### Case study 1: 4DCMR

**FIG. 3.**
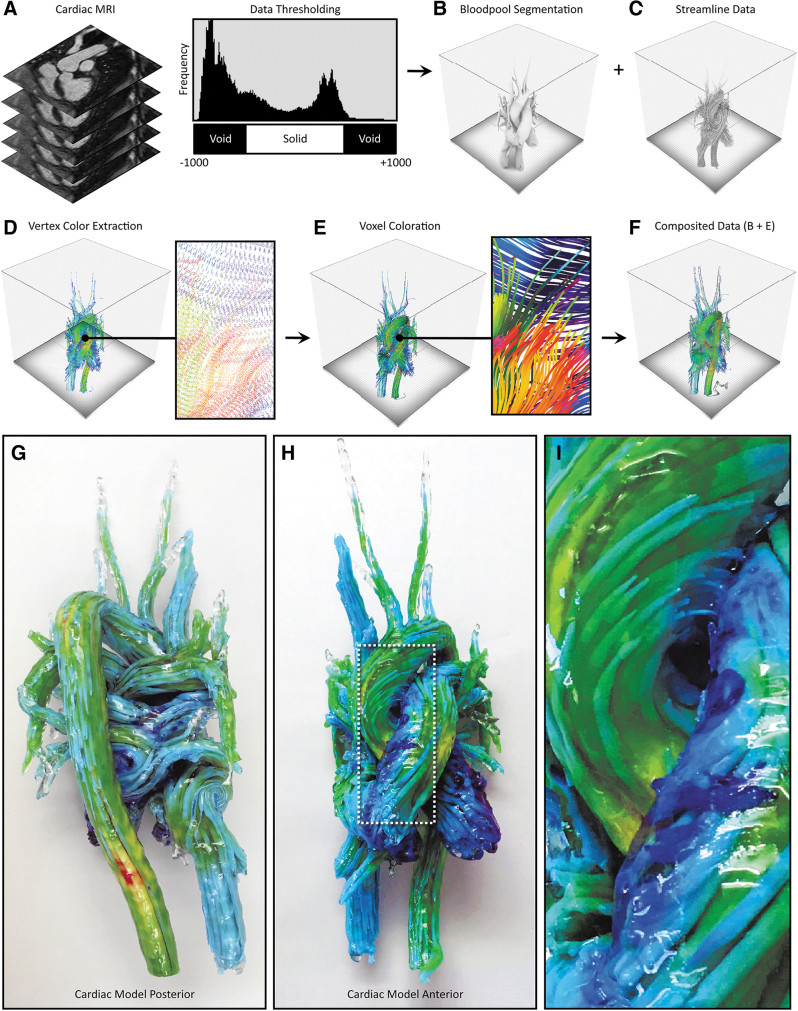
4D flow processing workflow and representative 3D-printed models from 4D flow data. From the input DICOM data **(A)**, the blood pool is segmented via an intensity value thresholding step to define the volume in which the streamline data are embedded **(B)**. **(C)** The PLY streamline data are then imported and overlaid upon the blood pool segmentation created in **(B)**. The PLY streamline mesh data are subsequently deconstructed to extract the color information stored in the vertices **(D)**. **(E)** The PLY mesh is next infilled with voxels that inherit the color of the nearest vertex to achieve a final specified streamline thickness. Finally, the streamline color data are composted with the blood pool segmentation and supersede the pre-existing color data from the background volume **(F)**. **(G–I)** A 3D-printed model of a 4D cardiovascular magnetic resonance scan of blood flow through a healthy adult heart, with streamlines visualizing the flow direction, which are color-coded based on velocity. 4D, four-dimensional.

**FIG. 4.**
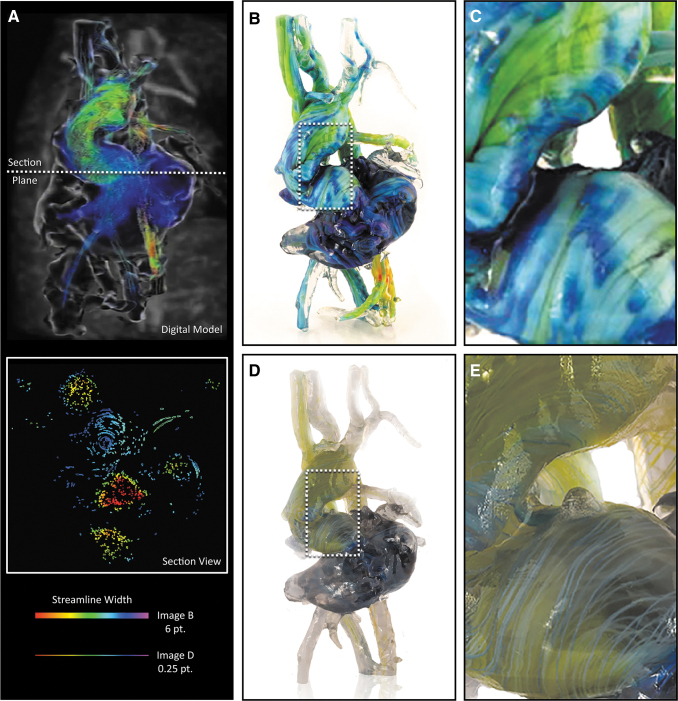
Exploring trade-offs between color saturation and data resolution during 4D flow visualization in 3D-printed models. Here, we explore the blood pool geometry of a 4.5-year-old child exhibiting dextrocardia with atrial situs solitus, an imperforate tricuspid valve, a double inlet LV, a large VSD, a double outlet right ventricle, a hypoplastic RV, malposed great vessels, a right aortic arch status post-Blalock–Taussig shunt, and a main pulmonary artery division with a bidirectional Glenn. **(A)** A digital computational model of 4D flow from a single frame in the cardiac cycle, 30% diastole (upper), and a cross-sectional slice of the digital model demonstrating the interior geometry of the streamlines, and the related streamline thicknesses for each respective model in B, C and D, E (lower). **(B)** Photograph of a bitmap-printed model from the same digital file to clearly illustrate the large-scale variation in flow velocity (streamline diameter: 6.0 pt.). **(C)** Magnified view of the 3D-printed model shown in **(B)**, with the streamlines in this example globally adjusted to maximize color saturation. **(D, E)** Photograph of a bitmap-based 3D-printed model, where streamline thickness was adjusted to clearly visualize the individual streamlines throughout the entire printed volume. Despite the increased spatial resolution of this model compared with the one shown in **(B)** and **(C)**, the streamline diameter (0.25 pt. thickness) is such that variation in color is barely discernible. LV, left ventricle; RV, right ventricle; VSD, ventricular spetal defect.

Two 4DCMR data sets were utilized to demonstrate the versatility of our method (Figs. 3 and 4). The first data set was acquired from a healthy adult, whereas the second was from a 4.5-year-old child with a rare congenital heart defect. Both patients provided the required informed consent for publication, and surgeons were consulted throughout the data processing process to ensure that the features of interest were accurately conveyed in the 3D-printed models.

Similar to the methods described by Dyverfeldt et al.,^12^ digital 3D streamline models were created and processed with the Circle Cardiovascular Imaging Cardiac MRI software (CircleCVI)^27^ and exported using the Polygonal File Format (PLY), a mesh-based model that incorporates vertex color. In this multistep process, the cardiac blood pool was first segmented to produce a single homogeneous solid object using a thresholding mask, which was applied to the original MRI image stack from which the 4D CMR data were generated. The streamline PLY data were then overlaid on the solid blood pool model, where information about color, streamline diameter, and cross-sectional shape (e.g., circular, triangular, square) could be modified.

The PLY surface mesh file was next converted to a Volume Database (VDB) file, hierarchically storing voxel data in a tree-like data structure with similarity (Museth, 2013) to B+trees, where a volume was created by filling the streamline boundary surface with RGB-colored voxels at a resolution determined through explicit controls to match that of the 3D printer. This process involved the creation of a distance field to determine the specific location of each voxel relative to the position of its corresponding mesh vertices in the PLY file.

The generation of the image slice stack from the region of interest (ROI) was completed in a manner similar to that employed by Hosny et al.^25^ This method utilized a fully open source approach, resulting in a computationally low and a comparably fast process for slice generation. Models that utilize a singular data source, or in our case, two data sources where only one model contains graded volumetric data, benefit from this method due to the ease of dithering. In this process, the composited 3D model was first subdivided into 30-μm-thick data blocks. A virtual camera was then centered above each sliced data block, at a height that corresponded to half the radius of the ROI. Then, a PNG image file was saved for each data block (corresponding to a single data slice) from the virtual camera at a resolution dictated by the 3D printer's specifications. Each of the resulting 32-bit raster images was then dithered using a local perceptual algorithm to quantize the pixels into separate material droplet descriptions, each corresponding to a specific resin color within the 3D printer.

For the case of the heart model shown in Figure 4, the perceived streamline color saturation was directly related to their corresponding thickness, and to best illustrate this point, two different versions were produced by varying the isosurface offset controls, one with a thickness of 6 pt, and one with a thickness of 0.25 pt, to demonstrate the trade-offs between local feature resolution and global color saturation (Fig. 3B, E). As shown in Figure 3B, streamlines with a diameter of 6 mm produced a vivid visualization of color, but these thicker lines decreased the visual depth of the model.

By contrast, streamlines with a diameter of 0.1 mm were visually perceptible as lines; however, differences in color were barely discernible, or appeared muted (Fig. 3E, F). These trade-offs, between linewidth and color saturation (which can be easily tuned by the user), must be taken into account for the visualization of streamlines and balanced with the visual depth requirements for each patient in a case-by-case manner. Recent advances in the development of transparent colors, however, are leading to solutions to mitigate issues related to the oversaturation, allowing for the ability to achieve transparency without requiring dithering with transparent materials.

### Case study 2: DWI white matter tractography with fMRI

Our second case study involved a neuroimaging data set containing acquisitions for T1-weighted MRI, DWI, and fMRI. This retrospective data set (acquired from open source sample data) was acquired from a 6-year-old child with an eloquent-area brain lesion. Since all imaging was de-identified and not used clinically, the requirement for individual informed consent was waived.

**FIG. 5.**
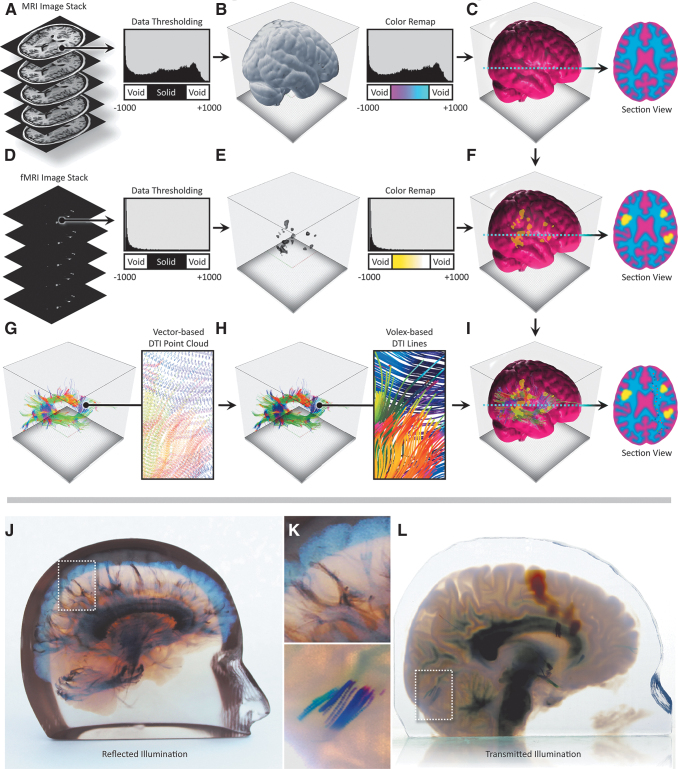
Neurosurgical multimodal data processing workflow and representative 3D-printed models from volumetric and vector-based data sets. **(A)** A T1 MRI data set is masked and thresholded to isolate the brain tissue and visualized as a volume rendering **(B)**. From the resulting masked brain data shown in **(B)**, user-defined colors are placed at luminosity values relating to the specific density ranges of gray and white brain matter **(C)**. **(D)** Using our image-based data processing method (Fig. 1D), bitmap image-based fMRI data are loaded and thresholded to ensure imaging noise is eliminated. User-defined colors are then placed at luminosity values to highlight variations in the intensity of the signal **(E)**. **(F)** The fMRI data are next overlaid on the T1 MRI **(C)**, and background color data are superseded by that obtained from the fMRI. **(G)** A vector-based diffusion tensor imaging tractography-based PLY model is loaded and deconstructed to extract color data from its vertices. The PLY mesh is infilled with voxels, which inherit their colors from their nearest vertex **(H)**. **(I)** The resulting vector-based data are overlaid upon the composted image data **(F)** and again, the background color data are superseded by that of the tractography data. **(J–L)** 3D-printed examples resulting from this data compositing method, using only *black*, *white*, and *clear* resins **(J)**, or in full color **(L)** are shown along with magnified views to reveal additional detail **(K)**. fMRI, functional magnetic resonance imaging; MRI, magnetic resonance imaging.

fMRI is an imaging technique to detect areas of the brain, which is active at a given time and was used in this case to clearly label the area responsible for speech near the brain lesion (Fig. 5D–G). BOLD imaging is a common method to gather fMRI data since it can be acquired on conventional MRI scanners.^28^ In our multistep process, the fMRI data were first imported as an 8-bit DICOM stack of 2D monochrome images, similar to the MRI data processing workflow described for case study 1. The resulting fMRI volume (Fig. 5E) was overlaid upon the whole-brain MRI data set (Fig. 5B) with reference to a pre-established color channel hierarchy, where the fMRI color channel replaced that of the MRI geometric data, to visualize the fMRI features of interest clearly.

In this process, the fMRI intensity values were remapped to colors through a user-defined lookup table that was chosen to emphasize the active regions. Because of the complexities of surface and subsurface scattering as noted in Bader et al.,^22^ our data processing workflows did not initially predictively translate into a representative physical object. An iterative process was therefore employed to calibrate our digital visualizations with the 3D printed physical object, which proved valuable for all subsequent 3D prints.

DWI is a noninvasive medical imaging modality that relies on the diffusion of water molecules inside tissues for the visualization of neurological structures.^29^ DTI is the output from a mathematical operation on an 8-bit DWI image stack and computes the distribution and major orientation of gradient directions in each voxel of the target tissue. Parameters that are derived from tensors in DTI can be visualized as 256-bit scalar voxel data, with vector field information consisting of X, Y, Z, and i, j, k values. These DTI tractography fiber data can be visualized and stored as a VTK file, which consists of a defined number of lines, where each line contains a unique identifier index value. Each of these lines consists of a number of spatial points that hold information about principal diffusion gradients, each of which is saved as a 3 × 3 tensor unit containing three eigenvectors and its integrated directionality, which are used to color-encode the fiber lines.

To illustrate this workflow, a 170 image, 256 bit T1 MRI data set with 1 mm isotropic resolution of a pediatric brain was first acquired from a Philips Medical Systems Ingenia scanner. The T1 MRI images were loaded into the 3D Slicer Medical Imaging Visualization^13^ platform to visualize the data as a 3D volume rendering. A skull-stripping machine learning-based algorithm was then employed to mask out bone from the DICOM images to isolate only the brain matter.^30^ The image stack resolution and slice thickness were extracted from the T1 DICOM metadata, and a voxel-based volume representation of the imaging data was created, defining morphology and density values in 3D space.

To clearly visualize the intracranial anatomy, the MRI data were then used to create a solid clear hull of zero thickness to enclose the T1 detail volume of the brain, similar to the methods described in Figure 5B. The T1 data were further enhanced using window/level adjustments to emphasize/de-emphasize specific features of interest and enhance changes in the density of the neural anatomy. The grayscale intensity values of the data were remapped to colors through lookup tables, which control color mixing and opacity ratios, similar to those described by Jacobson et al.^11^ Color and opacity were defined to allow for visualization of brain tissue, while allowing for embedded translucent objects of color to be visually coherent.

To efficiently slice through large numbers of tractography curves, we divided the curves into line segments and used an interval tree data structure to organize these segments and limit the number of intersection calculations per slice. The interval tree quickly retrieves all line segments that intersect a horizontal slice, taking into account line color and thickness. Once embedded within the brain voxel model, these curves can be voxelized at print time, allowing for the creation of very fine feature sizes throughout the volume, without the need to create computationally and memory-intensive meshes, as would be the case for an assembly of STL files. Once the curated T1 MRI, fMRI, and DTI tractography data are composited into a single digital model, and a *blue noise error diffusion dither*^31–33^ is performed volumetrically to quantize the six different printable resin colors (cyan, magenta, yellow, black, white, and clear) from material density values for each voxel. The end result of this multistep process is a quantized and dithered volume of material values that are then rasterized to a 32-bit PNG file for each printed layer, a description of which is shown in Figure 5.

## Discussion

The widespread adoption of 3D-printed models for presurgical planning has been a slow process, partly due to the inability to capture fine detail and material gradients with conventional surface mesh-based software data processing methodologies. The limitations of mesh-based 3D modeling of biological tissues have been widely documented and demonstrate fundamental limitations related to the lack of accuracy, complexity, and material gradients.^25,34,35^ In contrast, bitmap-based 3D printing approaches have proven to outperform and overcome many of these limitations for replicating patient-specific anatomy from medical imaging data.^36^

This method thus presents a valuable alternative to current practices by permitting the physicalization of anatomical and physiological complexities. In addition to 3D printing a representation of living tissue, which in contrast to conventional STL-based methodologies, can capture all the signal intensity gradients observed in traditional MRI and CT data sets; this bitmap-based 3D printing approach can be rapidly adapted to leverage the continually evolving range of available medical imaging modalities. Furthermore, our method demonstrates a significant improvement over traditional single data source-based bitmap printing methods^25^ by fusing disparate multimodal imaging data types into a single intuitive model.

### Lessons from case study 1: applications in cardiology

To demonstrate the value of our method for a cardiac application, we used CMR imaging data from a pediatric patient with tricuspid atresia, a rare congenital heart defect that affects ∼1 in 10,000 births in the United States. During an initial evaluation, and due to the very unusual orientation of the patient's aorta, it was suspected that blood flow through the ventricles and into the aorta would be highly abnormal and likely inefficient. To investigate this possibility, a 4D flow MRI analysis modeling technique was used to derive flow parameters, including measurements of wall shear stress, pressure difference, turbulent kinetic energy, and intracardiac flow components, and digital 3D anatomical and hemodynamic models of the patient's heart were created to assist in further clinical planning.

From these analyses, the bitmap-printed 3D model of the heart clearly illustrated the very unusual blood flow pathways and provided comprehensive hemodynamic information at each anatomical region during a single cardiac cycle. Further analysis of the 3D-printed model confirmed that the child's pumping efficiency would likely be improved after the Fontan procedure compared with patients with more typical tricuspid atresia. This bitmap-printed 3D model of 4D flow MRI analysis ultimately became part of the patient's permanent medical record, and as such, will continue to provide valuable information to direct future potential management changes and therapeutic planning in this specific, and related cases. Further clinical significance could be created by combining 4D flow with additional cardiac data sources such as shear wall stress and tissue compliance to provide a more holistic anatomical guide for surgical planning.

### Lessons from case study 2: applications in neurosurgery

The need for neurosurgeons to better understand the complex anatomy of the central nervous system is pushing the use of hybrid models combining morphological information from CT and MRI with physiological data from DWI, DTI, and fMRI.^37^ The inclusion of physiopathological data from advanced MRI sequences enables neurosurgeons to quantify metabolic activity within a specific lesion or identify a surgical pathway through complex neuroanatomical regions before surgery or biopsy procedures.^21^

**FIG. 6.**
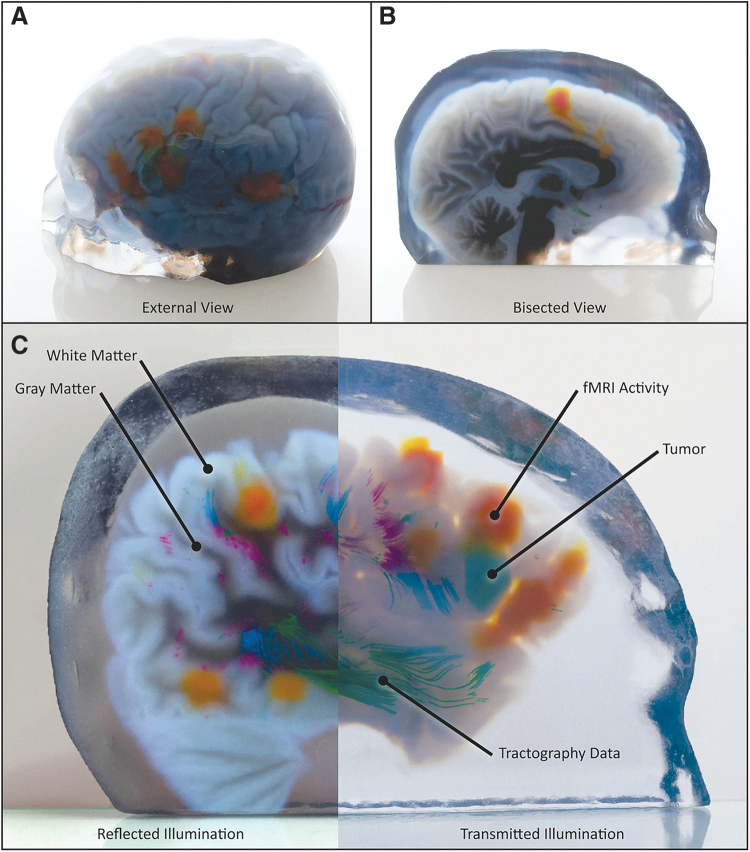
Representative 3D-printed models of multimodal medical data. **(A, B)** A bitmap-printed model of a pediatric patient with a Broca's area tumor is shown in *green*, fMRI data are shown in *orange*, and full-color tractography data from the arcuate fasciculus, viewed from the left posterior **(A)** and from an axial cross-section through the corpus callosum **(B)**. **(C)** A representative slab of the same model, photographed with either reflected (*left*) or transmitted (*right*) illumination to demonstrate variations in transparency and visual coherency of specific 3D features of interest (white matter, gray matter, fMRI activity, tumor, and tractography data).

The patient from case study 2 was diagnosed with an eloquent-area lesion centered in the Broca area. This brain region is located in the inferior frontal gyrus of the dominant cerebral hemisphere for language and is responsible for speech. Resection of a Broca's area tumor is a high-risk procedure because of the anatomical complexity and critical functionalities of the surrounding soft tissue that must be considered.^38^ Our bitmap-based 3D print of this specific case, which visualizes the tractography fibers (Fig. 6), depicts the arcuate fasciculus, a white matter tract that connects the Broca area and the Wernicke area in the brain. The Wernicke area is located in the posterior superior temporal gyrus of the dominant hemisphere and is responsible for the decoding and comprehension of speech.

A patient with Wernicke's aphasia is unable to understand speech, and while they are able to produce speech, it generally consists of intelligible words placed in an incomprehensible order. In contrast, those with damage to the arcuate fasciculus experience a specific deficit called conduction aphasia. They retain the speech production ability of Broca's area and the language comprehension of Wernicke's area, but are unable to repeat new words, and often make basic errors in pronunciation. The risk of damaging this language processing area can lead to Broca's aphasia, which is an inability for a patient to speak, and has a quality-of-life score on par with quadriplegia.^39,40^

Due to its local anatomical complexity and the large number of adjacent tracts, the arcuate fasciculus is often difficult to clearly visualize in relation to operative targets using conventional 2D viewing methods. Furthermore, function in Broca's and Wernicke's areas mapped via fMRI is often challenging to co-register with the DTI data that map the arcuate fasciculus due to the lack of available landmarks. An accurate and intuitive representation of the arcuate fasciculus in physical space relative to Broca's and Wernicke's areas, and a surgical target, would therefore greatly assist the surgeon in determining an operative strategy.

By integrating all the imaging technologies that are required for the unambiguous visualization of different aspects of cranial anatomy and physiology, our bitmap-based 3D-printed models represent valuable presurgical planning tools, enabling neurosurgeons to evaluate all the possible surgical approaches to preserve patient function while maximally reducing tumoral bulk, especially in deep brain locations.^15,20,41^

## Conclusions

With the methods described in the present study, we have shown that a variety of multimodal and correlative computationally derived physiological data sets found in medical imaging can be directly manufactured into physical models using bitmap-based multimaterial 3D printing. These approaches offer an improvement over current single data source bitmap-based 3D printing techniques and point toward new design opportunities for which the perceived barriers between the physiological and morphological domains can be obviated with ease through the fabrication of intuitive data-rich tangible objects. In the fields of electrode positioning for brain stimulation or radiofrequency techniques, for example, some authors have proposed the use of hybrid CT and MRI models to confirm the correct location for electrodes in deep brain stimulation, such as in treatments for Parkinson's disease and numerous forms of epilepsy.^42,43^

Moreover, recent advancements on the materials development front point to the opportunity for combining our data compositing approaches with biomechanically accurate material formulations for the production of tangible anatomical models that mimic not only patient specific dynamic physiology but also provide a mechanically informed haptic engagement that mimics the functionally graded properties of living tissue. As demonstrated here, the relationships between data visualization, computer science, digital fabrication, and medicine are fostering an information revolution with the potential for increased communication, connectivity, and interoperability of data—bringing form and function together to improve patient health.

## Data and Materials Availability

All data needed to evaluate the conclusions in the study are present in the article and/or the Supplementary Materials. Additional data related to this article may be requested from the authors.
